# Bone Marrow Aging and the Leukaemia-Induced Senescence of Mesenchymal Stem/Stromal Cells: Exploring Similarities

**DOI:** 10.3390/jpm12050716

**Published:** 2022-04-29

**Authors:** Paola Fernanda Ruiz-Aparicio, Jean-Paul Vernot

**Affiliations:** 1 Grupo de Investigación Fisiología Celular y Molecular, Facultad de Medicina, Universidad Nacional de Colombia, Bogotá 111321, Colombia; pfruiza@unal.edu.co; 2 Instituto de Investigaciones Biomédicas, Facultad de Medicina, Universidad Nacional de Colombia, Bogotá 111321, Colombia

**Keywords:** BM aging, MSCs senescence, leukaemia, leukemic microenvironment, AML, B-ALL

## Abstract

Bone marrow aging is associated with multiple cellular dysfunctions, including perturbed haematopoiesis, the propensity to haematological transformation, and the maintenance of leukaemia. It has been shown that instructive signals from different leukemic cells are delivered to stromal cells to remodel the bone marrow into a supportive leukemic niche. In particular, cellular senescence, a physiological program with both beneficial and deleterious effects on the health of the organisms, may be responsible for the increased incidence of haematological malignancies in the elderly and for the survival of diverse leukemic cells. Here, we will review the connection between BM aging and cellular senescence and the role that these processes play in leukaemia progression. Specifically, we discuss the role of mesenchymal stem cells as a central component of the supportive niche. Due to the specificity of the genetic defects present in leukaemia, one would think that bone marrow alterations would also have particular changes, making it difficult to envisage a shared therapeutic use. We have tried to summarize the coincident features present in BM stromal cells during aging and senescence and in two different leukaemias, acute myeloid leukaemia, with high frequency in the elderly, and B-acute lymphoblastic leukaemia, mainly a childhood disease. We propose that mesenchymal stem cells are similarly affected in these different leukaemias, and that the changes that we observed in terms of cellular function, redox balance, genetics and epigenetics, soluble factor repertoire and stemness are equivalent to those occurring during BM aging and cellular senescence. These coincident features may be used to explore strategies useful to treat various haematological malignancies.

## 1. Introduction

Cellular damage is considered the origin of the opposed processes in which the gain (cancer) or loss (aging) of cellular fitness define the phenotypic and functional characteristics of the affected cells [1]. In fact, there are some intriguing similarities in the hallmarks of these two cellular processes, i.e., cancer and aging, in relation to the causes, responses, and the local and systemic consequences of cellular damage [1,2]. Genome instability and mutations, epigenetic alterations and transcriptional deregulation, telomere/telomerase dysfunction, and cell death or immortality induce adjustments in cellular physiology by which cells try to respond in order to re-establish proper functioning. Since this is, in general, only partially achieved, these genetically, phenotypically and functionally altered cells modify their microenvironment, causing additional important changes in the surroundings cells and in the way they interact with each other.

Cellular senescence is a physiological program with both beneficial and deleterious effects on the health of organisms in different homeostatic processes, and is considered to be an example of evolutionary antagonistic pleiotropy [3,4]. It is one of the various optional outputs that result from cells when they are subjected to intense intrinsic or extrinsic stress signals, inducing cellular and, in particular, macromolecular damage, that cannot be repaired by the classical repairing systems. There are no uniform phenotypes or unique cell characteristics of senescent cells after receiving the stress signals that generate them, and now, it is believed that this process is multi-step, evolving, diversifying, and self-adapting [5].

In the last decade, the knowledge and interest in both aging and cellular senescence and their connection has increased considerably. Taking into account that both processes are at the base of the development of different diseases, the aim of ongoing research is to develop novel strategies that could reduce or modulate cellular senescence in tissues, eventually decreasing the physiopathological effects of aging [6]. In fact, senescent cells have been found to accumulate with increasing chronological age, and are supposed to drive aging and several age-related diseases, including haematological cancers [1,7].

Aging of the BM is believed to be the cause of the increased incidence of haematological malignancies in the elderly, and this can be envisaged as a result of both age-associated haematopoietic stem cell (HSC) dysfunction [8] and the particular altered characteristics and functions of aging stromal cells building a permissive milieu where leukaemia can be established and developed [9]. There is already abundant evidence showing that leukemic cell growth induces important alterations in the bone marrow (BM) microenvironment and, in turn, this remodelled BM has important roles in leukaemia maintenance, protection, progression, drug resistance, and relapse [9,10,11,12]. Since haematological malignancies have different cells of origin, diverse genetic abnormalities and also present distinctive clinical manifestations, one would imagine that the interaction with the BM microenvironment (and their modifications) might be specific to a particular leukaemia subtype. This is true to a large extent, but it is also possible to find common elements altered in mesenchymal stem/stromal cells (MSCs) that can be exploited to develop therapies targeting these particular coincident features, thus widening their applicability.

Here, we will review the connection between BM aging and cellular senescence and the role these processes play in the maintenance and progression of leukaemia. Specifically, we discuss the role of MSCs as a central component of the BM supportive niche for leukemic cells and leukemic stem cells (LSCs). We place an emphasis on acute myeloid leukaemia (AML) and B-cell precursor acute lymphoid leukaemia (B-ALL) since, in elderly AML, there is enough evidence to suggest a role of aged BM in the initiation, progression, and complication of the disease. On the other hand, in B-ALL, a disease mainly of childhood, the aging of the BM seems not to be an issue, but recent evidence showing a leukemic-induced senescent process in MSCs that could modulate the disease suggests that common elements must be present in these two different leukaemias. We propose that MSC alterations create a modified microenvironment that reinforce, expand (in AML), and induce (in B-ALL) MSC senescence, suggesting that shared strategies and cellular mechanisms may exist in leukemic protection and progression. As the BM microenvironment also contributes to treatment failure, the modified characteristics and altered cellular programs in MSCs will also be discussed in the context of chemoresistance; this knowledge should also be useful to develop new therapeutic strategies.

## 2. Bone Marrow Aging and Cellular Dysfunctions

BM aging is associated with perturbed haematopoiesis, imbalanced differentiation, vascular remodelling, altered immune response, inflammaging (a low-grade and persistent chronic inflammation and a ubiquitous characteristic of aging tissue), and propensity to haematological transformation [13,14,15,16]. Aging is associated with changes in the number and function of HSCs [17] due to cell intrinsic alterations such as DNA damage and epigenetic dysregulation [18]. In advanced/extreme stages, haematopoiesis could evolve into clonal haematopoiesis and/or HSC exhaustion. The former could have pathological consequences if a high number of driver mutations are present [19,20]. Moreover, aged BM secretome activates a pro-inflammatory program contributing to HSC clonogenic impairment [21]. HSC functions and integrity rely also on the support given by a dynamic BM niche made up of various cell types including MSCs, pericytes of mesenchymal origin, endothelial cells, osteoblasts, and diverse mature haematopoietic cells, among others [11,22,23]. In aged BM, HSC are relocated away from endosteal vascular niches with a reduction in self-renewal capacity, quiescence, and entry into the cell cycle [17].

Early and abundant work has shown decreased differentiation capabilities with increased BM age, in particular, with regard to osteoblastic lineage [24,25,26]. Aging induces the loss of mineralized bone (increasing the risk of osteoporosis) and a reduction in angiogenic factors [27]. There is also an important increase in adipocytes, which have a negative effect on bone formation (due to increased numbers of osteoclasts and augmented bone resorption), and consequently on HSCs [28,29]. This balance between osteogenesis and adipogenesis, affecting HSC function, depends mainly on the environmental cues sensed by MSCs [30,31].

During aging, the BM vasculature shows both morphological and metabolic changes with a significant reduction in arteriolar vessels [16]. In experimental mouse models, it has been shown that aging reduced the niche-forming vessels in the skeletal system, imposing the drastic remodelling of the BM vascular architecture with a deterioration of arteriolar structure [32,33]. In particular, the reduction in type-H endothelium (or transitional vessels) causes a decline in HSC maintenance and quiescence factors, reducing HSC frequency and impairing long-term repopulation activity [32,34,35]. The aged BM vasculature shows increased leakiness with exposure to blood plasma components, inducing high levels of reactive oxygen species (ROS) in proximity to HSCs and a decreased expression of the main HSC maintenance factors, as well as C-X-C motif chemokine ligand 12 (CXCL12) and stem cell factors (SCFs) in MSCs, with a concomitant increase in HSC differentiation and migration [36,37,38].

In aging, decreased functionality of the immune system has also been reported, increasing susceptibility to infections, the development of autoimmune disorders, and haematological malignancies [16,39]. Immunosenescence of the adaptive immune system also contributes to inflammaging and the deregulation of the cell-mediated immune response [40]. The inflammaging-induced activation of stromal and immune cells produces the secretion of interleukin-1 (IL-1), IL-6, IL-8, TNF-α, and others [41]. These cytokines initially modulate the immune response but, if the stimuli persist, as occurs in chronic inflammation during aging, then deleterious effects are observed with important consequences on HSCs and their progenitors, including permanent modifications of the stromal compartment. This hampers the ability of BM to support and maintain stem and progenitor cells with significant changes in other BM resident cells [42]. An important source of inflammaging is cellular senescence, a response to cellular damage and stress [43,44]. Age-related decreases in lymphoid potential and function, probably due to HSC relocation to the central niches and bone loss, have been described and could be responsible for myeloid lineage bias in adulthood [45,46]. The pro-inflammatory cytokine milieu is likewise a characteristic of haematological malignancies.

As if that were not enough, primary dysfunction of BM cell components could initiate secondary haematological neoplasms [13]. In fact, several experiments performed in mouse models have shown that some haematological malignancies can originate from BM microenvironment alterations [47,48,49,50,51,52]. Preliminary evidence has shown that these findings could be extended to human BM niches where specific inflammatory signals could induce genotoxic stress in heterotypic stem/progenitor cells that might be relevant in leukaemia predisposition disorders [53]. Additionally, there is evidence that an aged BM microenvironment favours the growth of clonally expanded haematopoietic cells having some somatic mutations (pre-leukemic cells) that could be responsible for AML establishment [54]. This issue, although appealing, will not be treated here; however, readers are invited to refer to recent excellent reviews on this topic [11,55,56].

## 3. The Aging and Senescence of Mesenchymal Stem Cells

As mentioned above, haematopoietic niches are composed of diverse types of cells, of which MSCs, in spite of their heterogeneity [9,11,57], have acquired much attention due to their capacity to differentiate into mesenchymal lineages (osteoblasts, chondroblasts, and adipocytes), to stimulate bone growth and remodelling, and their ability to support haematopoiesis [58,59]. Confirming their capability to support and maintain HSC properties, MSCs are capable of ectopically organizing HSC niches with fully functional haematopoiesis [58].

Cellular senescence is generally defined by changes in cell morphology, senescence-associated β-galactosidase (SA-βGAL) activity, and a characteristic growth arrest that is fuelled by the Rb/p16 and/or p53/p21 pathways [43,60]. It is supposed to be a cellular response to limit the proliferation of damaged and pre-tumour cells [43]; nevertheless, its participation in the progression of cancer has also been duly demonstrated [61,62,63,64]. Senescent cells secrete various pro-inflammatory cytokines, chemokines and growth factors, and proteases (the senescence-associated secretory phenotype or SASP) [63] that modify neighbouring cells, maintaining and extending senescence and inflammation, conferring plasticity to tumour cells, and thus severely altering the microenvironment [65]. Senescent cells accumulate in tissues; however, different organs display particular levels of senescent markers as a function of age across the human life span [6,66]. BM is no exception, with old MSCs being four-fold more positive for SA-βGAL activity and showing reduced proliferation with extended cell population doubling time, the upregulation of the p53 pathway, and cells with anomalous morphology [67].

As research progresses in this area, it has become more complicated to find distinctive age-induced changes in MSCs. It is known that these alterations depend on multiple variables, including the range of age groups chosen for the comparison, the species, the gender, the tissues studied, and other variables [68]. Yet, it is clear that BM interactions with senescent MSCs, as part of the whole aging of the organism, have a clear impact on the development and progression of age-related diseases, including haematological ones. This is due, in part, to the decline of or alterations in MSC proliferation, SASP, differentiation and regenerative potential, and increased cell exhaustion, among others [69,70,71,72]. In turn, different stressors, including genetic instability, telomere attrition, ROS production, mitochondrial dysfunction, irradiation, oncogenic activation, some chemicals, among other causes, can induce MSC senescence [1,73]. Although, aging per se and senescence induced by extrinsic stressors impinge specific properties to MSCs, the existence of common MSC alterations in AML and B-ALL would suggest that they use the same molecular mechanisms to remodel the BM microenvironment.

## 4. The Effect of Leukemic Cells on Mesenchymal Stem Cell Properties

Haematological cancers growing within the BM can induce modifications in the microenvironment, altering cell behaviour and function in different ways. This is the case of various myeloproliferative neoplasms [74], myelodisplasic syndrome [75,76], AML [77], and ALL [78], among others. Various cells of origin and diverse genetic abnormalities are at the base of these different diseases, and therefore progression and response to therapy vary considerably depending on the manner by which these leukemic cells establish a relationship with the supportive BM niche. On the other hand, similarities may also exist that can be used to understand the mechanisms of disease progression and eventually establish general strategies to control it. In the following sections, we will present and discuss MSC alterations in two different (in origin, presentation, progression, and severity) leukaemias: first, adult AML characterized by a hierarchical structure of cell population, with an altered primitive haematopoietic stem cell, the LSC, at the origin of the bulk leukemic cell population with the capacity of a leukemic initiating cell (LIC) [79]; second, B-ALL, mainly a childhood disease that is thought to arise in committed-B-lineage cells [80] and differs from AML in that all bulk cells have the capability of LICs, and therefore there is no clear hierarchical organization of the leukemic cell population.

AML has a higher incidence and worse prognosis than other subtypes of leukaemia (ALL, chronic myeloid leukaemia and chronic lymphoid leukaemia) [81]. AML is typically a disease of older adults (>65 years old), with 10 times more incidence than in younger people [82]. Patients typically present peripheral pancytopenia with a concomitant reduction in normal myeloid and lymphoid lineages in the BM where the leukemic blast population dominates cell growth. This suggests a direct pathophysiological connection between leukemic cell growth and the disruption of normal haematopoiesis [77]. This would appear to be not simply an unequal competition between leukemic blasts having advantages in cell growth compared to normal haematopoietic cells, but instead a situation in which instructive signals from the leukemic cells are delivered to MSCs in the BM, fostering their growth to the detriment of normal haematopoiesis. In recent years, a high number of new mutations of AML have been discovered that alter haematopoietic progenitors and induce malignant transformation and clonal expansion [83]. Since LSCs have self-renewal properties and show cell-cycle quiescence with the capacity of disease maintenance in the long-term, disease progression and relapse should be more related to the properties of LSCs than to non-LSCs [84,85]. Nevertheless, LSCs are not only very infrequent, but also heterogeneous [86]. Therefore, revealing how they instruct MSCs is a difficult task, and final MSC modifications should undoubtedly represent the effect of the bulk AML cells.

B-ALL is predominantly a childhood disease, although it can occur in youth and adulthood. Clinical outcomes have improved notably in recent decades, with >80% survival rate in children [87]. B-ALL symptoms include bleeding, fatigue, and infections. The clinical presentation, response to treatment, and outcome depend principally on the genetic and molecular defects of the leukemic blast [88]. B-ALL includes multiple subtypes defined by structural chromosomal alterations with secondary somatic mutations, DNA copy number alterations, and specific sequence mutations that contribute to leukaemogenesis [88,89,90]. Genomic analysis in B-ALL has been fundamental for diagnosis, risk stratification, treatment monitoring, and targeted-therapy development. Evidently, this last issue has taken into account the cell-intrinsic features of the leukemic cell, but recent research has shown that leukemic cell-extrinsic factors (stromal cells, soluble factors, etc.) are key determinants of leukemic growth, maintenance, and response to therapy. In fact, alterations of the BM microenvironment and BM MSCs are also seen in B-ALL with, for example, a characteristic loss of CXCL12 [91]. As with other haematological malignancies, B-ALL cells instruct MSCs to direct support in favour of the leukemic cells while affecting diverse MSC functions and normal haematopoiesis. As mentioned, the hierarchical model of leukemic growth does not seem to be applicable in B-ALL, with all leukemic blasts being capable of inducing leukaemia in experimental models (LIC function). Compared to AML, the information related to MSC alteration in B-ALL is minimal, as is knowledge of the molecular mechanisms involved in these modifications.

So as to facilitate the recognition of common and relevant aspects of MSCs related to aging/senescence vis à vis leukemic growth, we have separated, somewhat arbitrarily, the different topics to be addressed, as follows: (1) cellular dysfunctions, (2) redox balance, (3) genetic, epigenetic and gene expression, (4) SASP and inflammaging, and (5) stemness properties. Finally, we discuss how the knowledge of MSC modification can be used to increase drug sensitization. Although the available information on each topic is dissimilar, interesting coincidences are revealed.

### 4.1. Cellular Dysfunctions of MSCs in Aging and Senescence

While the cell surface markers classically used for MSC identification (CD44, CD73, CD90 and CD105) are relatively stable without minor or inconsistent variation with age [73], MSC morphology is severely affected during aging, with the appearance of longer and flattened cells that are positive for SA-βGAL activity [1]. Aging induces changes in MSC adhesion molecules (VCAM-1, vascular cell adhesion molecule 1, and CXCR4, C-X-C motif chemokine receptor 4) that alter migration, homing capacities and HSC support [92,93,94,95,96,97]. In another study, a significant reduction in both the expression of CD146 (MCAM, melanoma cell adhesion molecule) and in cells positive for this marker was detected in aging [21]. Functional annotation clustering revealed that aged MSCs have an affected cytoskeleton organization due to lower actin turnover, which leads to inferior responses to biological and mechanical signals [98,99]. Though somehow controversial, most studies have reported a progressive decline in colony-forming unit fibroblasts (CFU-F) in MSCs, and again a reduced proliferation rate with age [21,98,100,101].

Additionally, during prolonged in vitro culture, similar modifications and changes in gene expression are revealed. MSC morphological changes, with the increased expression of genes that codify for focal adhesion and actin cytoskeleton organization, have been shown [94]. A recent systematic review has revealed an increase in SA-βGAL activity in senescent MSCs, particularly in the expression of p53, p21 and p16, Rb, ROS, and NF-κB, together with decreased proliferation markers [102]. The prolonged expression and activity of any of these regulatory components is sufficient to induce senescence [4], initially reducing the proliferation rate until the characteristic cell growth arrest of senescent cells is reached [70,103,104].

Protein degradation involves the interconnected autophagy process and ubiquitin-proteasome system and their imbalance can induce the accumulation of protein damage and cellular senescence [105,106,107]. Both aging and senescence have been associated with decreased proteasome function [108,109,110,111]. MSC senescence involves a reduction in the expression of some proteasome subunits, a detrimental effect on proteasome activity, and an accumulation of oxidized proteins [112,113,114]. The reestablishment of the proteasome function allows the MSC’s lifespan to increase, and preservation of stem cell function [114].

Acute stressors can induce MSC senescence through a reduction in autophagy; the inhibition of autophagy induces MSC senescence, showing that both processes are connected [112,115]. This detrimental effect alters MSC stemness properties [116,117,118], and autophagy reestablishment can rescue MSC function [119,120,121,122]. There are also a few studies suggesting that increased autophagy favours a senescent state in MSCs [123,124,125], suggesting that autophagy can exert both pro- and anti-senescence effects [126,127], depending on the autophagy type and cell damage or stress [73]. The fact that senescent MSCs and aged MSCs share some properties lets us suppose that there are common signatures in chronological and cellular aging. Indeed, it has been reported in MSCs that a relation between gene expression profiles of age-induced changes and replicative senescence may exist. This implies that the molecular effects of aging in vivo and senescence in vitro have similar mechanisms [70].

#### 4.1.1. Cellular Dysfunction of MSCs in AML

AML MSCs are almost unaffected in their expression of classical cell surface markers: they have slight variations in CD44, CD73, CD90 and CD105 expression, and are negative for CD34 and CD45 [77,128]. Early work has shown that MSCs isolated from AML patients showed an impaired capacity to support normal haematopoiesis [129,130]. Moreover, MSCs suppress the cell apoptosis of in vitro-cultured primary AML cells; thus, favouring leukemic functions over those of HSCs [131,132]. It is believed that both direct contact and soluble factors secreted by MSCs are responsible for the leukemic pro-survival effect of MSCs. Adhesion molecules and cytokines playing a critical role in niche retention were altered in AML MSCs: a reduction in SCF, thrombopoietin, angiopoietin-1, and VCAM-1 [77,133], and an increase in CD146, integrin-α5 and CXCL12 [130,133] were shown. In particular, CD44 and very late antigen-4 (VLA-4) receptors expressed in AML cells were found to be essential for MSC adhesion [134]. In experimental mouse models, it has been shown that VLA-4 blocking antibodies are able to reduce minimal residual disease and favour AML survival [135]. CXCL12 is just one of several soluble factors (see below) constitutively expressed by MSCs that can stimulate AML cell survival [136,137]. Targeting its CXCR4 receptor (by antagonist or neutralizing antibodies) reduced AML burden and disease progression without affecting HSC engraftment [138]. Not all of the survival mechanisms induced by MSCs have been totally elucidated, but it is suggested that common adhesion downstream effectors are responsible and may be effective targets of treatment [139]. AML-derived MSCs showed diminished cell growth and CFU-F, and altered morphology [77,140,141,142]. The gene ontology analysis of healthy MSCs co-cultured with primary AML cells also showed the reduced proliferative capacity of MSCs [140].

The inhibition of autophagy seems to have an anti-tumour effect on AML [143,144]. Little is known regarding the modulation of this mechanism in MSCs by AML cells, but an increase in MSC autophagy was observed, with autophagy-related 5-protein upregulation. Accordingly, its inhibition induced alterations in MSC differentiation, cell cycle arrest, and an increased chemosensitivity of AML cells, with a parallel reduction in the expression of CXCL12 [145]. The simultaneous knockdown of ATG7 in MSCs and AML has increased AML susceptibility to chemotherapy, compared to only AML cell inhibition [146]. The above results suggest that autophagy modulation is reciprocal, and target strategies must contemplate the role of the stromal support.

#### 4.1.2. The Cellular Dysfunction of MSCs in B-ALL

The classical cell surface markers CD44, CD73, CD90, and CD105 were found to be normally expressed or slightly increased in MSCs co-cultured with the leukemic B-ALL cell line REH [147,148]. Here, also, MSCs induce leukemic cell survival with cell apoptosis and the inhibition of in vitro-cultured primary B-ALL cells [149]. An important upregulation in the adhesion molecules VCAM-1, ICAM-1, and VLA-5 was detected in this leukemic niche. Additionally, MSCs looked bigger, flattened, and vacuolated, with a reduced proliferation rate, and finally showed cell cycle arrest after few days. In addition, malignant B-ALL cells may modify the BM microenvironment by generating signals targeting stromal cells. In a xenotransplantation B-ALL model, it was shown that leukemic SCF production suppressed the CXCL12 expression of MSCs, affecting their HSC supportive function [150]. The CFU-F assay showed lower colony formation in MSCs, and as a validation of the results obtained in the in vitro co-culture system, all these changes were also observed in MSCs isolated from B-ALL patients [148,151], showing a clear impairment of MSCs in the ability to support CD34 progenitors [56]. In a B-ALL experimental model, it was demonstrated that leukaemia induced minor changes in MSC numbers, although MSC progenitors were increased while mature MSCs were reduced [152]. Interestingly, it has been shown that, in the late leukemic stages, mature MSCs adopt a transcription signature similar to progenitors.

Evidence of autophagy in the MSCs of B-ALL cells is scarce; however, it has been described that B-ALL cells transfer autophagosomes to MSCs in a tunnelling nanotube (TNT)-dependent manner. How this mechanism alters MSC function is unknown; nevertheless, taking into account the autophagy role in cytokine signalling and inflammation, the authors suggest that this phenomenon could explain the release of factors exerting a supportive effect on leukemic cells [153].

### 4.2. The Redox Balance of MSCs in Aging and Senescence

The redox balance in cells is well regulated by the antioxidant enzymes that are highly expressed in MSCs [154]. The fact that deregulation in autophagy is linked to the susceptibility of MSCs to oxidative stress and mitochondrial dysfunction [155] is not surprising, because the greatest contribution to ROS production originates from the electron transport chain in the mitochondria [156]. Differences in structural and functional characteristics of the mitochondria have been observed in aging MSCs and in in vitro-cultured MSCs. These changes were found to be accompanied by an increase in ROS production [26,157], and were the main cause of mitochondrial dysfunction, inducing defects in complex I and III, exacerbating ROS production and accelerating MSC senescence by an increase in p53 and p21 expression [107,158]. After several in vitro passages of MSCs, mitochondria mis-localization increases ATP content and decreases oxygen consumption and NADH levels [159,160].

It has been suggested that ROS accumulation and mitochondria dysfunction in MSCs could be responsible for a metabolic change in oxidative phosphorylation (OXPHOS) towards glycolysis [161,162], alleviating oxidative stress [163]. On the contrary, other authors have shown that aged MSCs have a lower glucose metabolism, and that ATP production in senescent MSCs is mainly by OXPHOS [112,160]. Although controversial, this would demonstrate a metabolic shift towards OXPHOS concomitant with senescence, linked to the loss of metabolic flexibility [112].

Increased oxidative stress, inducing extensive macromolecular damage, especially in DNA and proteins (oxidized, misfolded, denatured), drives a full senescent program in aged MSCs [99,164]. DNA damage accumulates with age, mainly due to telomere attrition after replicative stress and loss of fitness in the DNA repairing mechanisms [165]. DNA lesions trigger DNA damage response (DDR) pathways and cell cycle arrest to allow DNA repair. If the damage is severe, exceeding repair efficiency, MSCs may establish either senescence or apoptosis programs [165]. Persistent oxidative stress induces a rapid phosphorylation and co-localization of ATM, γH2AX, and 53BP1, leading to DDR through the activation of the p53 and p38MAPK pathways, both responsible for the cell cycle arrest of MSCs [166]. Importantly, ROS-stressed MSCs decrease their capacity for proliferation, differentiation, immunomodulation, and cell support [167].

Other cellular physiology features revealed in in vitro cultures are much more difficult to evaluate in aged BM MSCs, but it is quite possible that they might occur in vivo. For example, in 3D cultures, senescent MSCs were capable of transferring cytoplasmic content to non-senescent MSCs via TNTs, with a consequent reduction in p16 [168], probably driven by oxidative stress, since mitochondrial damage can induce TNT formation [169].

#### 4.2.1. Redox Balance of MSCs in AML

AML MSCs showed increased levels of ROS and oxidative stress, and the nuclear translocation of transcription factors associated with the expression of antioxidant enzymes [170,171]. These alterations induce modifications in MSC behaviour, allowing the transfer of mitochondria to the AML cells [170,172,173,174]. Interestingly, NADH-oxidase-2-dependent superoxide production in AML cells can drive the mitochondrial transfer [170] and induce a senescent phenotype in MSCs that is modulated by p16 and SASP [175]. Contra-intuitively, the mitochondria uptake by AML cells did not increase their ROS production, due in part to the antioxidant effect exerted by MSCs and dependent on reduced glutathione and glutathione peroxidase activity [176]. Nevertheless, MSCs improved the bioenergetic capacity of AML cells by increasing OXPHOS potential and ATP production [176]. In this sense, the expression of reduced glutathione-related antioxidant genes is linked to poor prognosis in AML [176].

AML MSC senescence may also be driven by the higher expression of p21 and p53 and increased SA-βGAL activity [128,140]. Of note, a feedback loop between long-lasting DDR and increased ROS production is needed for the development and maintenance of senescence [177]. These findings show that AML MSCs tend toward senescence, as has been shown in vivo in AML patients [175].

#### 4.2.2. Redox Balance of MSCs in B-ALL

In addition to the metabolic adjustment of leukemic cells, MSCs are affected metabolically during co-cultures. A simultaneous change from OXPHOS towards aerobic glycolysis was observed in both cell populations. The MSC metabolism modulated by B-ALL induced an impaired response to oxidative stress and mitochondrial respiration [178]. This induced the release of MSC lactate, which is an important intermediary to sustain leukemic cell survival [178,179].

Leukemic cell lines induced in cultured MSCs a transient ROS production in cytoplasm and mitochondria [147]. The SA-βGAL enzyme was also increased with a concomitant expression of either the p53 or p16 pathways (depending on the leukemic cell line), whose activation has been linked with persistent cell stress and cell cycle arrest in aging cells [147,180]. This, together with the above-observed changes in MSCs, confirms that contact of B-ALL cells with MSCs induces in the latter an early senescence process. This is comparable to MSCs isolated from B-ALL patients at diagnosis [148]. An accelerated senescence process in MSCs has also been described for T cell-ALL [181]. It was shown that ROS production was sufficient to induce DNA damage and the DDR. Additionally, a small proportion of MSCs co-cultured with REH cells for 3 days were positive for γH2AX [148]. Since MSCs, like other stem cells, have efficient mechanisms of DNA repair, this DNA damage was corrected after the further co-cultivation of cells for a few days, suggesting that the senescence process was transient, as was ROS production. In fact, it was demonstrated that, after leukemic cell removal, MSCs restored their original morphology, re-entered the cell cycle, reduced SA-βGAL activity, and acquired normal functioning [148].

**Figure 1:**
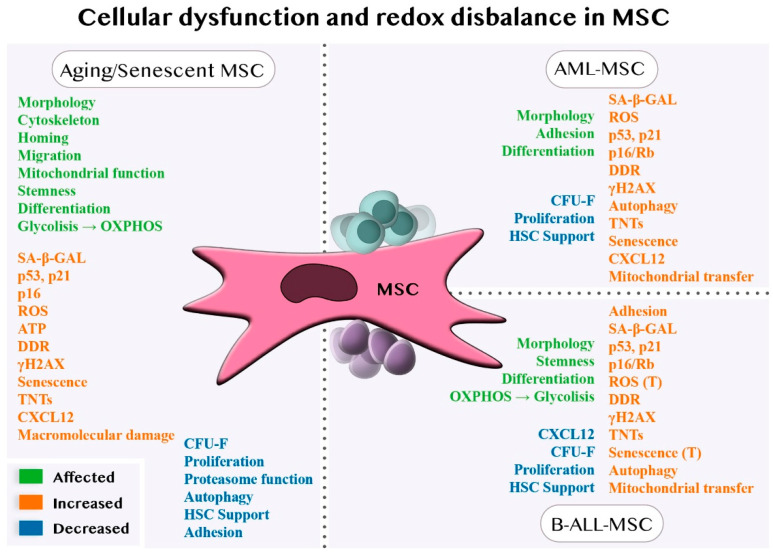
Cellular changes in MSCs induced by aging/senescence and hematological malignancies (AML or B-ALL). MSCs subjected to a physiological aging process or exposed to a pathological environment in the presence of leukemic blasts exhibit common characteristics. MSCs enter senescence, overexpressing molecular markers related to DNA damage and cell-cycle arrest. At the same time, they showed impairment in functions associated with HSC support, the maintenance of the redox balance, and homeostasis. In B-ALL, some of these characteristics seem to be transient (T).

To conclude Section 4.1 and Section 4.2, both aging and leukaemia induce morphological changes in MSCs with altered adhesion molecule expression that affects MSC intrinsic characteristics such as homing or migratory capacities and hematopoietic cell support. These MSCs have increased SA-βGAL activity, an increased expression of genes associated with cell cycle arrest, ROS, SASP, and DDR, and reduced proliferation and CFU-F. These factors, along with mitochondrial dysfunction and metabolic alteration, induce and reinforce the senescent process (Figure 1).

### 4.3. Genetic, Epigenetic and Gene Expression Alterations of MSCs in Aging and Senescence

Heterochromatin disorganization is considered a driver of MSC senescence in aging and disease [182,183]. Cellular models of aging display enlarged nuclei, signs of nuclear DNA decondensation, and heterochromatin loss [184]. Interestingly, lamin B1 downregulation has been shown to be a key trigger of global and local chromatin reorganization during cell senescence, having an impact on gene expression and aging [185]. Within the local heterochromatin reorganization, it is very likely that those driving the expression of SASP genes (key effectors of the senescence program) must be present.

Epigenetic modifications in aging or replicative senescence alter gene expression, MSC number, and stemness properties, suggesting that both processes are similar and/or linked [73]. The trimethylation of histone H3 (H3K9me3), which induces the strong binding of the histone complex to DNA and forms heterochromatin, is gradually lost during the aging process. H3K9 demethylases plays an important role in the regulation of DDR and senescence. Senescence-associated heterochromatin foci increase in senescent cells, silencing genes involved in cell division [186]. Nevertheless, senescent cells still experience global heterochromatin loss, with the subsequent expression of genes that were previously inaccessible to the gene expression machinery [184]. Thus, despite the increase and redistribution of localized facultative heterochromatin, the chromatin changes observed during senescence also seem to support the heterochromatin loss model of aging. During MSC senescence and aging, DNA methyltransferases expression was found to be significantly downregulated [187]. Aging MSCs have three times more hypomethylated than hypermethylated autosomal CpG sites [188], which is in agreement with the general data of DNA methylation on aging [189].

The long-term culturing of MSCs is associated with DNA methylation changes at specific promoter regions, which then become either hypermethylated or hypomethylated [97]. DNA hypermethylation increases in genes related to the regulation of DNA replication, cell cycle, DDR, multipotent differentiation, and metabolism [190]. Additionally, it has been found that, in in vitro-cultured MSCs, there has been a gradual decrease in global DNA methylation, with some coincidences in these changes aligning with aging MSCs [191,192,193]. In another study, it was found that cultured MSCs had decreased level of the histone methyltransferase Ezh2, showing the involvement of histone modifications in cellular senescence [194].

Comparison of the gene expression from human BM MSCs isolated from young and older donors showed, in the latter, the upregulation of 67 genes and the downregulation of 60 genes [70]. Among the upregulated genes, those involved in the extracellular matrix regulation, mesoderm development, proliferation and chemotaxis were found [70]. In this study, it was remarkable that several age-related gene expression changes in MSCs were also differentially expressed upon in vitro replicative senescence.

#### 4.3.1. Genetic, Epigenetic and Gene Expression Alterations of MSCs in AML

Some early work has shown the presence of cytogenetic abnormalities in MSCs from AML patients [195] and, in another study, it was found that about 16% of MSC samples from AML patients presented genetic abnormalities that were different from leukaemia blasts [196]. This was also confirmed in another study [197].

Although the role of epigenetics has only begun to be explored in the leukemic niche very recently, some important keys have been revealed. A decreased expression of the chromatin remodelling complex CHD1 (modulating chromatin condensation) in AML MSCs has been determined. This CHD1 reduction is associated with the loss of CFU-F and HSC supportive capacity [198]. On the other hand, the methylation status of multiple CpG sites and islands was examined in the MSCs of AML patients: 228 hypermethylated CpG site probes covering 183 gene symbols, and 523 hypomethylated CpG site probes covering 362 gene symbols were identified [199]. In other study, AML MSCs revealed global hypomethylation compared to controls [130]. This is a consistent feature of human cancers, in which the specific hypermethylation of CpG islands occurs concomitant with global hypomethylation [200].

In a recent study, it was revealed that the protein expression in AML MSCs was different from normal MSCs. The presence of AML cells induces changes in the transcriptional profile of MSCs, downregulating cell-cycle-related genes and supporting a high expression of cytokine-related genes [140]. Additionally, it was shown that the distinct expression patterns of MSCs were characteristics of AML patients and associated with a heterogeneous clinical outcome, suggesting that BM MSC remodelling may serve as a prognostic factor. In another work, reverse-phase protein array analysis showed the differential expression of 28 proteins between AML MSCs and normal MSCs, that were then used to define four different MSC populations in AML MSCs [128]. Interestingly, patients having one (with relevant integrin/GSK3 axis) of these four groups of cells presented longer survival and remission duration compared to other groups of patients having other MSCs classes. The fact that MSCs with a particular phenotype and function have clinical relevance suggests that MSC modulation could favour disease outcome.

#### 4.3.2. Genetic, Epigenetic and Gene Expression Alterations of MSCs in B-ALL

MSCs isolated from B-ALL patients have shown a relative genetic stability. For instance, the typical translocations present in the leukemic clone, or other chromosomal abnormalities developed in in vitro cultures, were not found in MSCs, even after chemotherapeutic treatment [201]. Other authors, studying MSCs in B-ALL patients with different genetic rearrangements, found only a small percentage of MSCs that acquired the KMT2A-AF4 fusion gene [202]. To our knowledge, no epigenetics changes in MSCs have been reported in B-ALL.

Set enrichment analyses have shown that MSCs co-cultured with B-ALL cell lines augmented the expression of different sets of genes related to epithelial–mesenchymal transition, inflammatory response, and TNF-α signalling via NF-κB, changes that may participate in niche remodelling [203]. The role of NF-κB seems relevant since there is a reciprocal activation of NF-κB signalling in MSCs and the B-ALL cell line REH in co-cultures [204], and genome-wide analyses of these MSCs have revealed the increased transcription of genes related to this factor. We also have described that NF-κB activators are overexpressed in MSCs after contact with ALL cells, at the same time that the expression of the inhibitors IκBα and IκBε were reduced [205]. In a similar setting, it was demonstrated by microarray analysis that the main cytokines produced in this leukemic niche, and targets of NF-κB signalling, were IL-6, IL-8, and C-C motif chemokine ligand 2 (CCL2) [147].

The upregulation of the serine-threonine kinase C isoform PKC-βII in MSCs was also demonstrated after the binding of leukemic cells to MSCs, and it was proposed that this PKC activation could be a standard mechanism to co-opt MSC survival signals [206] and that it could be used to control leukaemia. In fact, blocking specific signalling molecules in MSCs (Erk, p38, PI3K/Akt, PKC, and others) has been suitable for this purpose [149,203,207,208,209,210,211,212].

### 4.4. SASP and Pro-Inflammatory Stimulation in Aging and Senescence

In agreement with the establishment of a senescence program in aged MSCs, an increase in the pro-inflammatory SASP was detected at the transcriptional and protein levels [21]. This collection of soluble factors [63,213] is connected to beneficial as well as deleterious effects of senescence. Nevertheless, SASP composition varies according to the senescence inducer, the time of induction (acute or chronic senescence), the cell type, the cell neighbourhood and, importantly, the context in which senescence occurs [4,68,214]. Therefore, it is extremely difficult to anticipate the precise composition and physiological effects of SASP. In spite of this, among the key components of SASP, IL-6 and IL-8 are the most conserved and robustly expressed cytokines [215,216,217]. The persistent secretion of SASP allows the establishment of a low-grade long-lasting inflammation state called inflammaging that reinforces senescence in a cell autonomous fashion and can be transferred to healthy surrounding cells (paracrine stimulation), amplifying both senescence and inflammation [44]. This could also happen by ROS transfer through gap junctions during cell interactions and the further ROS-mediated activation of NF-κB signalling [73,218,219], the major intracellular pathway directing SASP production. Other signalling pathways responsible for SASP production, involving p38MAPK and mitochondrial dysfunction, have been described [220,221]. Through these signalling pathways, senescence can be definitively established, contributing to the deterioration of physiological fitness with age.

It has been suggested that chronic inflammation drives HSC myeloid skewing and leads to HSC exhaustion during aging [222]. An important role in this process has been assigned to CCL5 and IL-6 [223,224]. Additionally, the BM accumulation of adipocytes during aging contributes to myeloid-biased differentiation and reduced B lymphopoiesis [225,226].

Extracellular vesicles (EVs) exert an important role in MSC communication with their surroundings, and have been proposed as a non-canonical part of SASP [227,228]. Aged or senescent MSCs increase EV production [227,229,230], which can either relieve the stress or spread the pro-senescent effect [227,231]. Indeed, EVs from young MSCs restored the cell growth of aged MSCs, reducing senescence and increasing pluripotency [230].

#### 4.4.1. SASP and Pro-Inflammatory Stimulation in AML

The pro-survival effect of MSCs on AML cells is dependent, in part, on the soluble factors secreted by the MSCs. The pro-inflammatory genes IL-1β, IL-8, CXCL1, CXCL2, CXCL3, and CCL2 were the most upregulated in MSCs co-cultured with AML leukaemia cells [10]. Other authors have found downregulation of the SCF and the overexpression of Jagged1 in human AML MSCs [77]. In a recent study, it was found that the secretion of higher concentrations of IL-10 by BM MSCs correlated with a short survival of AML patients [197], showing a link between in vitro MSC modifications and response to treatment, thus having clinical relevance. Since MSCs are diverse and functional heterogeneity has been demonstrated [128], it is believed that the secreted soluble factors will also be different. However, if one considers that a senescent process is taking place in BM MSCs, and that the autocrine and paracrine effect of SASP will predominate after definitive senescence establishment, SASP could eventually be the dominant assortment of secreted factors present in the microenvironment. AML cells induced an increase in IL-6 production by MSCs, correlating with disease progression [232]. IL-8 seems to be especially relevant in AML, where it can be secreted by AML cells upon interaction with CXCR1-expressing MSCs that are capable to migrate in response to it [233]. The interaction of AML cells and MSCs also promotes the production of IL-8 by MSCs, conferring an advantage to the survival of AML cells [234,235]. In this manner, IL-8 production is reinforced in paracrine and autocrine ways.

On the other hand, it seems that the differential secretion effect of MSCs depends on the stemness attributes of the AML cells. For instance, BM MSCs co-cultured with leukaemia cell lines had different degree of stemness (CD34+CD38− or CD34+CD38+ or CD34− cells) upregulated differentially with the expression of sets of genes. AML cells that showed less stemness properties were able to upregulate a number of pro-inflammatory cytokines (IL-8, CCL2, CXCL1, IL1β, through IL17, CD40 and NF-κB signalling), while AML cells that were more stem-like upregulated IRF8-related genes [10].

#### 4.4.2. SASP and Pro-Inflammatory Stimulation in B-ALL

The main pro-inflammatory cytokines secreted in an in vitro leukemic niche of MSCs with REH cells were IL-6, IL-8, CCL2, CXCL1, and CCL5 [204,236,237], which are well-known NF-κB targets. These cytokines were not secreted by the individual cells (MSCs or B-ALL cells), but were the consequence of the cell interactions. In other studies, patient-derived pre-B-ALL cells or leukemic cell lines induced the secretion of CXCL10, IL-6, IL-8, CCL2, and CCL5 [203,238]. Especially, CCL2 and IL-8 enhanced the adhesion capacity of B-ALL cells to MSCs and stimulated the proliferation and survival of MSCs, reinforcing the MSCs’ support capacity [237]. Consistent with the fact that SASP secretion increases the expression of adhesion molecules [239], the strong binding of B-ALL cells to MSCs is evident after co-culture, a phenomenon that must contribute to MSCs’ protective effect. The activation of a pro-inflammatory program seems to exert a key role in leukaemia progression [56,203]. Additionally, IL-6, IL-8 and CCL2 are increased in children at B-ALL diagnosis [237,240], showing their relevance in vivo. A pro-inflammatory milieu was evidenced during leukaemia growth, with IL-6 being one of the major secreted cytokines and an important participation of IL-7 in disease progression [152]. Other genes were downregulated in MSCs derived from ALL patients, such as CXCL12, IL-7 and the B cell-activating factor BAFF, which are important molecules in B cell physiology [55].

Although the evidence of EVs derived from ALL MSCs is limited, it has been shown that galectin-3, present in MSC EVs, favours its own expression in AML cells with a reduced therapy sensitivity in AML cells via Wnt/β-catenin signalling [241]. Interestingly, TNT formation stimulated the production of CXCL10, CXCL8 and IL-2 by MSCs co-cultured with B-ALL cells [238].

### 4.5. Stemness Property Modifications of MSCs in Aging and Senescence

It has been shown that, with age, human MSCs lose their multilineage differentiation capacity and have reduced clonogenic frequency [242,243]. MSCs from older subjects exhibit lower differentiation capacity and self-renewal properties, due in part to ROS and metabolic stress [73,100]. At the same time, MSCs show an age-dependent lineage switch between osteogenic and adipogenic fates, favouring adipocyte generation over osteoblastic lineages, whose molecular mechanisms and physiological consequences are fairly well understood [9,244]. In other studies, aging showed a reduction in MSC proliferation and oteoblastogenesis with a downregulation of genes involved in osteogenic differentiation [67,96].

Cellular senescence is an intrinsic cell barrier for cell reprogramming or plasticity; however, both are less efficient in cells from old organisms due to the upregulation of the Ink4/arf locus (encoding three strong tumour suppressors) during aging [245]. However, it has been shown that senescence, through SASP production, can induce cellular plasticity and tissue regeneration [246]. During ex vivo MSC expansion, the expression of stemness-associated genes Oct4, Nanog, and Tert decrease [247]. In this sense, two transcription factors associated with stemness, Twist and Oct4, are able to block or reverse senescence by suppressing the expression of p21 (through the upregulation of DNA methyltransferases) or p16/p14 (through Ezh1-dependent inhibition) [194,248]. MSCs cultured with EVs isolated from latter-passage MSCs showed a reduction in their osteogenic potential compared to EVs from the early passage, with changes in the expression of related genes [229]. EV miRNAs, such as miR-183-5p, are increased in aging BM or oxidative-stressed BM cells and endocytosed by MSCs fostering a reduction in osteogenic differentiation accompanied with the induction of a senescence phenotype [249]. Therefore, senescence and stemness are also linked processes.

#### 4.5.1. Stemness Properties Modifications of MSCs in AML

In general, AML MSCs exhibited a reduced ability to differentiate osteoblastic lineage. This was confirmed by specific methylation changes affecting several pathways involved in cell differentiation and skeletal development [77]. Moreover, a decreased number of osteoblasts was found in AML patients and in experimental models of AML, supporting a disturbed multilineage differentiation potential of MSCs in AML [250]. In the study by Kornblau et al., AML MSCs at diagnosis showed a higher expression of some proteins related to osteogenic differentiation compared to those of AML MSCs at relapse, a clear indication that disease progression affects their osteogenic capacity [128]. Nevertheless, in another study, it was shown that osteogenic differentiation in AML was necessary to support blast growth, and this was accompanied by adipocyte differentiation [142]. The reduction in osteogenesis was also accompanied by an increase in adipocytes in the BM that was suggested to influence the cellular energetics and was shown to be necessary for the survival of AML cells [251,252]. By comparing MSCs isolated from normal donor or AML patients, it was found that the latter had increased adipogenic potential with superior ability to support the survival of leukaemia progenitor cells [253]. Furthermore, gene ontology and pathway analysis revealed that the adipogenesis pathway was dysregulated in AML MSCs.

#### 4.5.2. Stemness Property Modifications of MSCs in B-ALL

MSCs are able to form clonal mesenspheres with the ability to self-renew, and this assay has been used to evaluate this property. The basal capacity of sphere formation was reduced in MSCs from an in vitro leukemic niche established with REH cells [147]. Mesensphere induction with growth factors showed the increased sphere formation of MSCs obtained from a co-culture with REH cells. Later, it was shown that this enhanced sphere formation was not due to cell adhesion molecule upregulation or leukemic cell growth within the mesenspheres [148], suggesting alterations of the stem cell properties in the MSCs. This was confirmed by showing the reduced osteoblastic differentiation capacity of MSCs. Additionally, MSCs isolated from B-ALL patients showed also altered differentiation capabilities to mesenchymal lineages, with reduced osteogenic and propensity to adipogenic differentiation [148]. In an immunocompetent B-ALL model, it was shown that the number of osteoblasts decreased in the BM, accompanied by an increased activity of RANKL and subsequent osteoclastogenesis and bone resorption [254]. These findings are in line with what has been described in patients with B-ALL [78,255], and seem to be a generalized abnormality in other malignancies [256,257].

**Figure 2:**
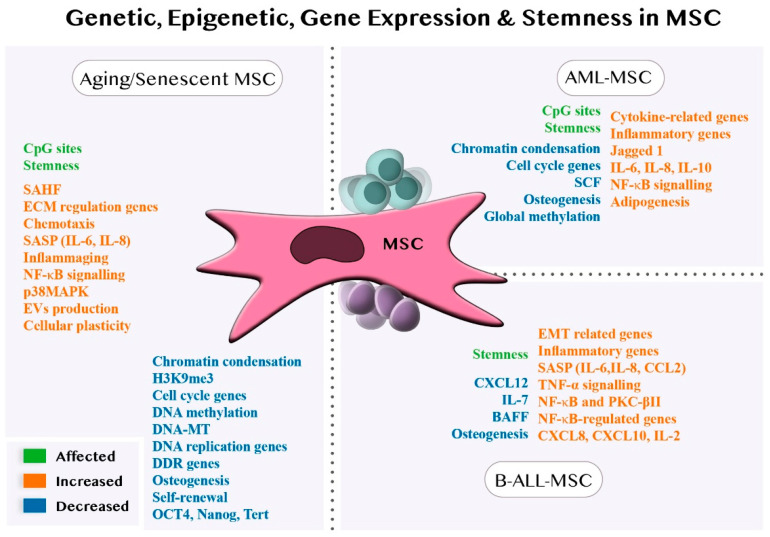
Expression profile, genetic, epigenetic and stemness-related changes in MSCs. Regarding the repertoire of soluble factors and cytokines, aged MSCs and leukemic MSCs show a proinflammatory signature mediated, in part, by NF-κB activation. Other genes related to replicative or proliferative capacities are repressed by changes in the epigenetic landscape, even though global DNA methylation is reduced. Stemness functions involving self-renewal, clonogenicity and differentiation potential are also impaired in both disease models. DNA-MT: DNA methyl transferases; ECM: extracellular matrix; EVs: extracellular vesicles.

Section 4.3, Section 4.4, Section 4.5 allow us to conclude that aged or leukemic MSCs present an altered gene expression program, with the induction of inflammatory and cytokine-related genes and altered stemness properties (reduced self-renewal and osteogenic differentiation and, in some instances, increased adipogenic differentiation) which affect the BM microenvironment concerning inflammation and senescence. Although a reduction in condensed heterochromatin predominates, specific regions of the genome associated with replication, repair, and the cell cycle are repressed (Figure 2).

## 5. MSC Roles in Drug Resistance

As shown above, MSCs are dysfunctional in patients with AML and B-ALL, exhibiting characteristics that are quite similar to those observed in aged or senescent MSCs. Of greater relevance is the expression of genes that produce an inflammatory milieu, increasing leukemic cell adhesion and protection. Additionally, the induction of oxidative stress, senescence, and SASP, producing further inflammation and senescence expansion, are characteristics of the reprogrammed MSCs. These complex MSC alterations should also play an important role in the drug resistance of leukemic cells and, therefore, may become interesting therapeutic target options. In fact, new research in this area is directed to develop antibodies or other compounds with the ability to interfere specifically with all the different supportive malignant cues (those originating from leukemic cells and/or from the leukemic niche). The great challenge is to find those that are common among different types of leukaemias (independent of the particular genetic lesion) and that may have a broader application.

### 5.1. Drug Resistance Mechanisms in AML

MSCs confer drug resistance to AML cells through a wide variety of mechanisms where the participation of the repertoire of soluble factors and cell interactions is well known [258]. These VLA-4/VCAM1-mediated interactions [204] promote AML cells’ chemoresistance by diverse mechanisms, including Bcl-2 overexpression [132] and JAK/STAT [259] and NF-κB signalling in MSCs. Thus, the inhibition of MSCs’ interaction with AML cells after CXCL12/CXCR4, CD44, ITG4 or E-selectin inhibition reduces the resistance of leukemic cells to chemotherapy [260,261,262,263,264,265]. The interaction between MSCs and AML cells induces a side population phenotype in leukemic cells which becomes more resistant to anthracycline treatment [266]. This resistance was acquired by stroma-dependent ABC drug transporter activation. Connexins (CX) also participate in intercellular communication and their expression is altered in aged MSCs [267]. CX are responsible for ROS transfers between cells in the BM microenvironment [268]. In particular, CX43 is an important regulator of autophagy and mitochondrial integrity [269]. Therefore, CX inhibition in MSCs induces an increase in the sensitivity of AML cells to chemotherapy [270].

In de novo-diagnosed AML patients, MSCs upregulated the expression of VEGF, IL-6, CXCL12, and indoleamine 2,3-dioxygenase, and reduced the expression of IL-10. AML MSCs showed a decline in the proliferative capacity, in part due to the IL-32 production that protects AML cells from cytarabine [271]. Of note, this profile was associated with bad prognosis in AML patients [272], suggesting that the secretory phenotype of MSCs could exert an important role in the course of the disease. A detailed network participating in AML resistance has been described [258].

More recently, the role of EVs was also included in the list of mechanisms contributing to AML cells’ protection [273]. EVs from MSCs and AML cells can reshape the BM microenvironment, and it has been demonstrated that EVs are increased in AML patients’ plasma, even after chemotherapy and remission [139,274]. RNAs, miRNAs and transcripts of FLT3-ITD, nucleophosmin-1 (NPM1), IGF-IR, CXCR4 and MMP9 present within EVs, alter stromal cells, and have important roles in AML treatment and prognosis [275]. TGF-β1, miR-155 and miR-375 from AML MSCs EVs protect AML cells against cytarabine and FLT3 inhibitors. MSCs respond to AML EVs by increasing IL-8 production, contributing to AML chemoresistance to etoposides [276]. Additionally, FGF2, secreted by MSC EVs, induces AML cell resistance to tyrosine kinase inhibitors, and reinforces the EV production in MSCs [277].

On-going clinical research, involving CXCL12, CXCR4 and IL-6 inhibition in combination with standard chemotherapy, is taking place for AML patients [278,279,280]. A better knowledge of how MSCs’ secretome, redox balance and stemness properties are affected would certainly allow the development of novel targets.

### 5.2. Drug Resistance in B-ALL

The importance of the BM microenvironment in ALL cell protection during chemotherapy is less well studied, but the fact that the disruption of this interaction can improve drug susceptibility in B-ALL and T-ALL suggests that it has a fundamental role [149,281,282]. B-ALL cell lines present differential adhesion to the stroma, with the most adherent cells exhibiting a quiescent state, altered metabolism, and less sensitivity to chemotherapeutic agents [283]. ALL cell interactions with stromal cells mainly involve CXCR4, VLA4 and CD44 [135,260,261]. In particular, VLA-4 has been identified as a risk factor in B-ALL [284], as its inhibition suppresses the activation of NF-κB in B-ALL cells [203]. Similarly, the activation of NF-κB in MSCs and leukemic cells contributes to B-ALL chemoresistance in VLA-4 and VCAM-1-dependent interactions [204]. Additionally, NF-κB inhibition improved the responsiveness of B-ALL to chemotherapy [203]. Nevertheless, when NF-κB inhibition was performed in MSCs before the co-culture with primary B-ALL cells, no sensitization to dexamethasone or vincristine was observed [149]. This could be due to the fact that NF-κB signalling is activated downstream of PKC-β, this enzyme being only expressed after B-ALL cell binding to MSCs [206]. The integrin- and PI3K-dependent signalling pathways ILK/Akt, ERK1/2, and STAT3 were activated in MSCs protecting leukemic cells from apoptosis in ALL and AML [285]. ILK can also be activated in leukemic cells, activating a feedback loop involving ILK/NF-κB, ending with CCL2 production and promoting leukaemia progression [286]. Growth factors and cytokines such as IL-6 produced by MSCs are able to protect leukemic cells from glucocorticoid-induced apoptosis by the upregulation of antiapoptotic molecules. In fact, when IL-6 production was inhibited in co-cultures, drug sensitivity was increased [287].

Notch-3/4 signalling pathways was also found to induce chemoresistance in B-ALL cells following corticosteroid treatment [288]. These molecules were overexpressed in leukemic cells and MSCs in co-culture conditions, and their inhibition reduced the viability of B-ALL cells. Chemoresistance effects were also found to be mediated by Jagged-1/2 through direct contact with MSCs. This alteration in Notch signalling by the co-culture is detrimental to the expression of markers typical of the osteogenic lineage, resulting in a remodelling of the microenvironment in vivo [289]. Under co-culture conditions, both MSCs and B-ALL cells can express high levels of WNT, which maintains leukaemia cell proliferation and cell cycle progression [290,291]. Treatment with a β-catenin inhibitor-sensitized leukemic cells both in vivo and in vitro to cytarabine treatment.

Another aspect that is relevant is the way in which the BM microenvironment can be modified by the treatment with different chemotherapeutic agents. For example, B-ALL cell lines upregulate the surface expression of the CXCR4 receptor, and its inhibition decreases the protection exerted by MSCs [292] with a reduction in tumour cell infiltration in vivo [293]. The elevated expression of CXCR4 in B-ALL blasts has been associated with poor prognosis [91,294]. In the presence of chemotherapeutic agents, leukemic cells can restructure the microenvironment by recruiting MSCs via the overexpression of CCL3, TGF-β1 and GDF15 [295].

It was recently shown that oxidative stress induces a senescent process in MSCs that increases the susceptibility of B-ALL cells to dexamethasone [148]. This should be of clinical relevance since chemotherapy itself induces senescence [296]. Other recent data propose a clinically useful mechanism for improving ALL treatment, as ROS-inducing chemotherapies are often ineffective at eradicating residual disease [173]. Oxidative stress phenomena can be induced in the stroma during the development of leukaemia [172] or by chemotherapeutic treatment [173]. MSCs contribute to the redox balance of leukemic cells by mitochondria transfer through TNTs. Importantly, a reduction in mitochondrial transfer by interrupting TNT formation with vincristine prevented the “rescue” function of activated MSCs [173]. MSCs also provide leukemic cells with metabolites such as asparagine by the glutamine exchange mechanism with ALL cells [297]. Additionally, the influence of the BM microenvironment induced an increase in ROS levels in ALL cells, but it also helped to alleviate this stress in an adaptative way, meaning that ALL cells were capable of upregulating their antioxidant defences. This redox adaptation process favoured ALL cells’ chemoresistance, which seems to be reversible [298].

Finally, an MSC-dependent effect on ABC transport upregulation was demonstrated in B-ALL cells subjected to dexamethasone [205]. This was evidenced by PKC inhibition in MSCs and in B-ALL cells still bound to MSCs, and it was found possible to dissociate the effect from the one produced by the inhibition of cell adhesion.

Although the knowledge acquired about the changes induced in MSCs by leukaemia has already been used for treatment, more work is necessary to untangle the way in which other alterations can be exploited to find extra tools to halt leukaemia growth. All other aspects involved in disease maintenance and evolution should be considered. In particular, alterations in osteoblastic and adipogenic differentiation or stemness functions should be further explored.

## 6. Concluding Remarks

The evidence presented here shows that there are many similarities between the changes that occur in MSCs in the BM as a result of aging and senescence, and those with induced leukemic growth of both AML and B-ALL. This, of course, does not rule out that there are many particularities in each of these processes (aging/senescence and leukaemia progression), and in each of these diseases (AML and B-ALL). However, we can summarize the following in relation to these coincident features.

Cell surface markers classically used for MSC identification are relatively stable without minor or inconsistent variation in age or senescence. Conversely, MSC morphology is severely affected during aging, with cells becoming positive for SA-βGAL activity and important changes in MSC adhesion and adhesion molecule expression occurring that alter their migration and homing capacities. Aged MSCs present a decline in CFU-F efficiency and cell proliferation, increases in p21, p53, and p16 expression, and have dysfunctional mitochondria, ROS production, and extensive macromolecular damage, collectively driving a senescent program. Some of the above-described features of MSCs are consistent with what we have stated here for AML and B-ALL.

Both aging and senescence are associated with decreased autophagy and proteasome function, similar to AML MSCs. To our knowledge, there is only one report of autophagosome transfer from B-ALL to MSCs, but its function is unknown. Nevertheless, in AML, a feedback loop between long-lasting DDR and increased ROS production allows the establishment of definitive senescence, while in B-ALL it seems that senescence is transient, although this was only demonstrated in vitro; in vivo, this could depend on other factors (blasts infiltration, disease state, treatment).

Heterochromatin disorganization drives senescence. As in other cancers, the specific hypermethylation of the CpG islands occurs concomitant with global hypomethylation in leukaemia. These changes alter gene expression with the important participation of NF-κB, are relevant to clinical outcomes, and are useful as prognostic factors. More research is needed to reveal the specific changes occurring in chromatin organization and epigenetics modifications during AML and B-ALL.

Aged MSCs show increased NF-κB activity and altered SASP, whose persistence may reinforce and transfer senescence to the healthy surrounding cells. This can be completed by ROS induction, DNA damage, and further NF-κB activation. This is also similar in AML and B-ALL. Nevertheless, a differential SASP depending on LSC stemness attributes has been demonstrated in AML. AML cells showing less stemness properties were able to upregulate a number of pro-inflammatory genes. A similar secretion profile induced by B-ALL cells in MSCs also involved NF-κB.

Several alterations occurring in MSCs are responsible for chemotherapeutic resistance in both AML and B-ALL. Therefore, blocking cell adhesion, soluble factors, and the intracellular signalling molecules responsible for these modifications are a complement to classical treatment. MSC modifications such as senescence induction, NF-κB activation, deregulated metabolism, propensity to SASP expression, and others, could be used as additional strategies to halt leukemic growth.

## Data Availability

Not applicable.
